# Spatiotemporally Detailed Quantification of Air Quality Benefits of Emissions–Part II: Sensitivity to Study Parameters and Assumptions

**DOI:** 10.1021/acsestair.4c00128

**Published:** 2024-08-27

**Authors:** Amir Hakami, Shunliu Zhao, Petros Vasilakos, Anas Alhusban, Yasar Burak Oztaner, Alan Krupnick, Howard Chang, Armistead Russell

**Affiliations:** † Department of Civil and Environmental Engineering, Carleton University, Ottawa, Ontario K1S 5B6, Canada; ‡ School of Civil and Environmental Engineering, Georgia Institute of Technology, Atlanta, Georgia 30331, United States; § Resources for the Future, Washington, D.C. 20036, United States; ∥ Emory University, Atlanta, Georgia 30322, United States

**Keywords:** Air quality, emission reduction, particular matter, health benefit, benefit per ton, CMAQ adjoint, sensitivity analyses

## Abstract

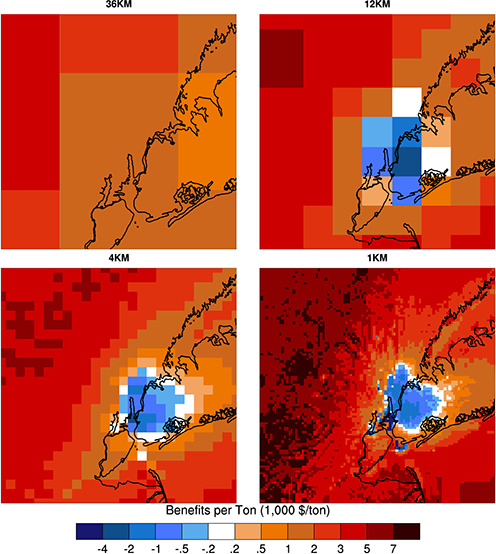

Adjoint modeling, using U.S. EPA’s Community Multiscale Air Quality (CMAQ), has been performed to provide location-specific monetized health benefits from the controls of primary PM_2.5_ and PM_2.5_ precursors (NO_*x*_, SO_2_, and NH_3_) across North America. Source-to-health benefit relationships are quantified using a benefit-per-ton (BPT) metric, accounting for the impacts on premature mortality due to long-term exposure to fine particulate matter. In the base analysis, the approach used a 12 km resolution, four 2-week episodes chosen to capture annual responses, emissions for 2016, and the Global Exposure Mortality Model (GEMM) to link exposures to premature mortality. Here, we investigate the impacts those choices have on results using a range of sensitivity analyses. The choice of four representative episodes led to relatively little bias and error. Finer model resolution, investigated by comparing 36, 12, 4, and 1 km simulations over two urban areas, tended to increase BPT estimates, though the impact was inconsistent between different regions. While BPTs and burden estimates were consistent across resolutions over New York City, they sharply increased for Los Angeles, particularly for NOx and ammonia, leading to 90% increase in burden estimates at 1 km resolution. We find that, for primary PM_2.5_ emissions, better resolved population distribution is the main contributing factor to higher BPTs, but for secondary precursor emissions (ammonia and NOx), higher model resolution that avoids dilution in coarser grids is more important. Changing emissions from 2016 to 2001 and 2028 resulted in fairly consistent primary PM_2.5_ BPTs but impacted the BPTs for NOx and ammonia more significantly due to changes in SO_2_ emissions. We found that BPTs tend to stabilize, as emission changes in 2028 lead to a lower deviation from 2016 BPTs compared to changes from the 2001 episode. The role of the epidemiological model also led to relatively modest uncertainties, 15–30% depending on the species, even when different shapes of concentration–response functions were employed. We found the impact of the choice of CRF to be larger or comparable in size to the reported epidemiological model uncertainties for log–linear CRFs. The adjoining approach proved robust to modeling choices in providing BPT estimates that are highly granular across locations and emitted species.

## Abstract 2

While modeling study design and assumptions give rise to uncertainties to varying degrees, location-specific benefits-per-ton (BPTs) from full-complexity model simulations remain robust to these inevitable uncertainties.

## 1 Introduction

One of the approaches used to attribute the population health burden of air pollution to contributing sources relies on the estimation of benefit-per-ton (BPT) metrics, calculated using various types of atmospheric models. BPT of an emitted pollutant is defined as the monetized societal benefits, or the avoided burden, associated with the reduction of a metric ton of emissions of the pollutant. Also referred to as marginal benefits, BPTs provide an easy and straightforward means for comparing the benefits of emission control options from various sources with each other or with the costs of emission reduction measures.

BPTs are often estimated for different emission sectors and geographic regions.^1−4^ Estimation of sectoral BPTs is a natural choice for regulatory reasons; however, aggregation of BPTs into regional estimates also stems from practical computational limitations. While estimating location-specific BPTs may be valuable, doing so using traditional techniques and chemical transport models (CTMs) is infeasible due to the computational demands of such calculations.^5^ One approach to address this limitation is to use reduced complexity models (RCMs) that employ simplified representation or approximation of atmospheric processes;^6−12^ the low computational cost of these models allows for estimation of location-specific BPTs or source impacts. An alternate approach that avoids the simplification of RCMs and uses a full-complexity representation of atmospheric processes is the use of an adjoint model for BPT estimations. Adjoint models^13−16^ can be used for reverse source influence modeling and relating population health burden to individual sources.^17−19^ Recently, Zhao et al. used the adjoint version of the U.S. EPA’s Community Multiscale Air Quality (CMAQ) model^16,20^ to estimate location-specific BPTs for Canada and the United States.^21^

Calculation of BPTs requires integration of some form of the atmospheric model with epidemiological concentration–response functions (CRFs) and economic valuation of societal impacts. Regardless of the approach used and the models involved, estimates of BPTs are subject to a range of uncertainties arising from various factors, such as inputs, model formulation and numerical solution, natural variability, or study design. Here, we conduct sensitivity analyses of location-specific BPT estimates with respect to various assumptions or study designs that are common in BPT studies. While this analysis is conducted for the BPT study of Zhao et al.,^21^ we believe different aspects of our analysis can be applicable to various other studies and approaches and can shed light on the limitations of BPT estimates in the literature.

## 2 Methods

There are a multitude of factors that can give rise to uncertainties in model-based source-impact estimates, such as BPTs. Here, we conduct sensitivity analysis on four specific aspects of the study design for BPT estimations for the U.S. We examine the sensitivity of BPT estimates to (a) episodic modeling of BPT estimates, (b) the choice and form of the epidemiological model, (c) spatial resolution of simulations, and (d) changes in emissions and atmospheric composition. While episodic simulations are more common for adjoint simulations, the issue of temporal representativeness (e.g., across years) is a general point of concern in source impact estimations regardless of the models or approach used. Other aspects of our sensitivity analyses are applicable to any form of BPT or other types of source impact assessment studies.

### 2.1 Modeling Data and Platform

**Figure 1:**
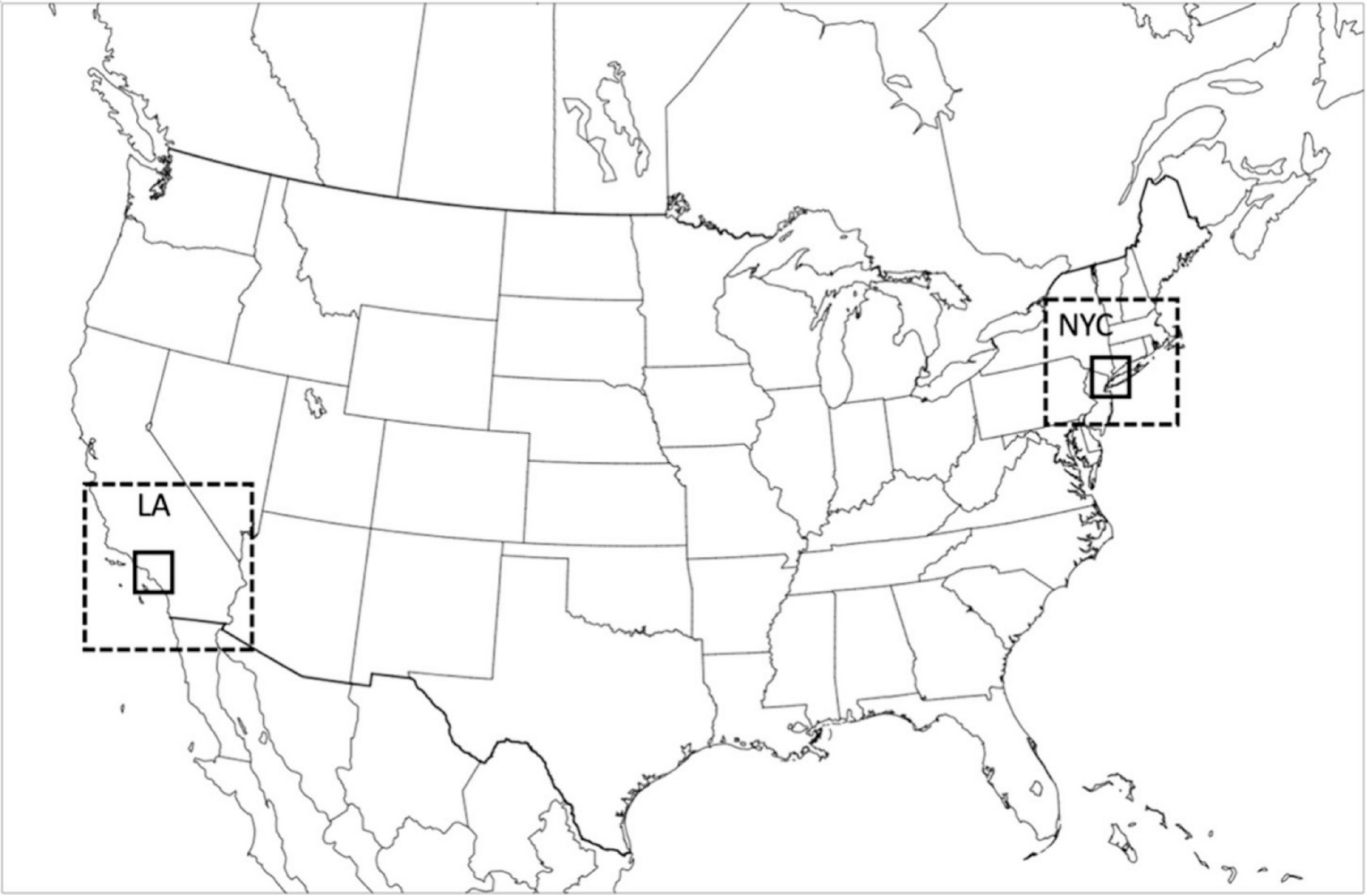
Nested domain for multiple resolution simulations for New York City and Los Angeles. The dashed and solid lines denote the 4 km and 1 km resolution domains, respectively, while the larger domain shown is for 12 km simulations.

We use CMAQ v5.0 and its multiphase adjoint to conduct our BPT simulations. Our adjoint simulations are conducted for seasonal episodes in the year 2016 and at 12 km horizontal resolution. We use the Global Exposure Mortality Model (GEMM)^22^ as our CRF of choice to estimate BPTs, while we conduct similar simulations with four other CRFs for sensitivity analysis. Our 12 km forward CMAQ and BPT simulations are informed by 36 km simulations at the initial time and lateral boundaries. We conduct additional 4 and 1 km simulations over NYC and LA for the summer of 2016 (Figure 1), as well as additional simulations with emissions inventories at 2001 and 2028 levels for sensitivity analyses.

We use meteorological data from the 2016 v7.2 platform that are based on the Weather Research and Forecasting model (WRF) version 3.8 at 12 and 36 km resolutions with 35 vertical layers.^23^ We also use WRF to conduct meteorological simulations for higher resolution domains that are not included in the 2016 platform. Emissions at all resolutions are based on the emission data from the 2016 platform.^24^ Emissions for the years 2001 and 2028 are processed using Sparse Matrix Operator Kernel Emissions (SMOKE)^25^ based on past or projected inventories. Age-specific population data for health impact assessment are at the census block level and aggregated to the CMAQ grid resolution. Similarly, baseline mortality rates for each CRF’s target population and health outcome designation are based on the Center for Disease Control’s baseline rate database at the county level and mapped/interpolated to the appropriate CMAQ grid resolution. Valuation of mortality outcome is based on the Value of Statistical Life (VSL) with income adjustment for the year 2016.^26^ Further details of the study can be found elsewhere.^21^

### 2.2 Sensitivity to Episodic Simulations

Computational cost of adjoint simulations renders year-long modeling at moderately high resolution infeasible. As such, episodic simulations are common in adjoint studies. Zhao et al. develop a method for systemic episode selection based on temporal bias minimization.^21^ They conduct yearlong adjoint simulations at a coarse resolution (36 km) and select the most representative two-week episodes for each calendar season that minimize the bias from seasonal BPT and BPT-based burden estimates across the continental U.S. These seasonal episodes are then used for higher resolution simulations (12 km at the continental scale and 4 and 1 km for sensitivity analysis; see below). The simulations at 12 km include ramp-up days and proper initial and boundary conditions from 36 km runs for both forward and adjoint models. To evaluate the impact of episode selection, we compare the BPT estimates based on full-year simulations with those constructed from episodic simulations at this coarse resolution with the implied assumption that temporal features at 36 and 12 km remain the same.

### 2.3 Sensitivity to the Epidemiological Model

The epidemiological model is a well-known source of uncertainty in any health impact assessment study, regardless of the methods, data, or models used. The uncertainties arising from the epidemiological models stem from statistical uncertainties (i.e., the goodness of fit or confidence intervals) in effect estimates but also from the shape of CRF when its form (e.g., log–linear) is prescribed a priori. BPTs depend on the slope of the population attributable fraction (PAF) which itself depends on the shape of the CRF, and therefore, in different concentration ranges, a CRF may result in smaller or larger BPT estimates than another (Appendix A, Figure A1).

We used a range of CRFs that have been previously used in the literature for PM_2.5_ chronic exposure mortality estimations. These include three log–linear, a sublinear, and a superlinear CRF. These CRFs include the following:

(a) Log–linear CRF of Krewski et al.^27^ based on the reanalysis of the American Cancer Society – Cancer Prevention Studies-II cohort (ACS-CPS-II), referred to as ACS-09,
(b) Log–linear CRF of Turner et al.^28^ (PM_2.5_) based on the extended ACS-CPS-II cohort (ACS-16),
(c) Log–linear meta-analysis CRF of Chen and Hoek^29^ (CHEN),
(d) Sublinear CRF of the Global Exposure Mortality Model (GEMM),^22^
(e) Superlinear CRF based on the National Health Interview Survey (NHIS).^30^

Further details of these CRFs, including their formulation, can be found in Appendix 1. While these 5 CRFs are used for epidemiological model sensitivity analysis, we primarily use GEMM for our BPT estimates and other sensitivity analyses.

### 2.4 Sensitivity to Spatial Resolution

To assess the impact of resolution on BPT estimates, we conduct additional 4 and 1 km simulations of two nested domains over New York City (NYC) and Los Angeles (LA) (Figure 1). Given the extreme computational cost of adjoint simulations at very high resolution, our resolution sensitivity analysis is only conducted for the summer season. We develop 4 km and 1 km emission inventories for these domains based on the same emission platform for the year 2016.^24^ We normalize emissions at higher resolution domains to match the aggregate emissions of the coarser grid cell they are part of to ensure emissions are consistent across all resolutions.

Our simulations over NYC and LA consist of four-level nested domains of 36, 12, 4, and 1 km resolutions. Progeny domains are informed by the lateral boundary conditions from parent domains for both forward and adjoint simulations. Boundary conditions for adjoint simulations ensure that the impact of sources on populations outside the higher resolution domains are captured and considered.^31^ The implementation of the backward boundary condition, a novel feature of our study, is essential to properly comparing BPT estimates across various resolutions.

### 2.5 Sensitivity to Emission Levels

BPTs are derivatives with respect to emissions and therefore should be viewed as slopes of the function describing monetized nationwide mortality. As slopes, BPTs are based on an implied assumption of linearity. In the presence of nonlinearity, significant changes in atmospheric composition, such as those driven by large-scale emission control measures across decades, can lead to increased errors in BPT estimates. To evaluate the robustness of estimated BPTs across decadal changes in emissions and North American atmospheric composition, we estimate the BPTs for past and future conditions. We conduct simulations for the years 2001 and 2028 for GEMM U.S. BPTs at 12 km and over two seasons (summer and winter). In these simulations, we use the same meteorology as that of 2016 to isolate the impact of changes in emission levels.

## 3 Results and Discussion

### 3.1 Episode Selection

**Figure 2:**
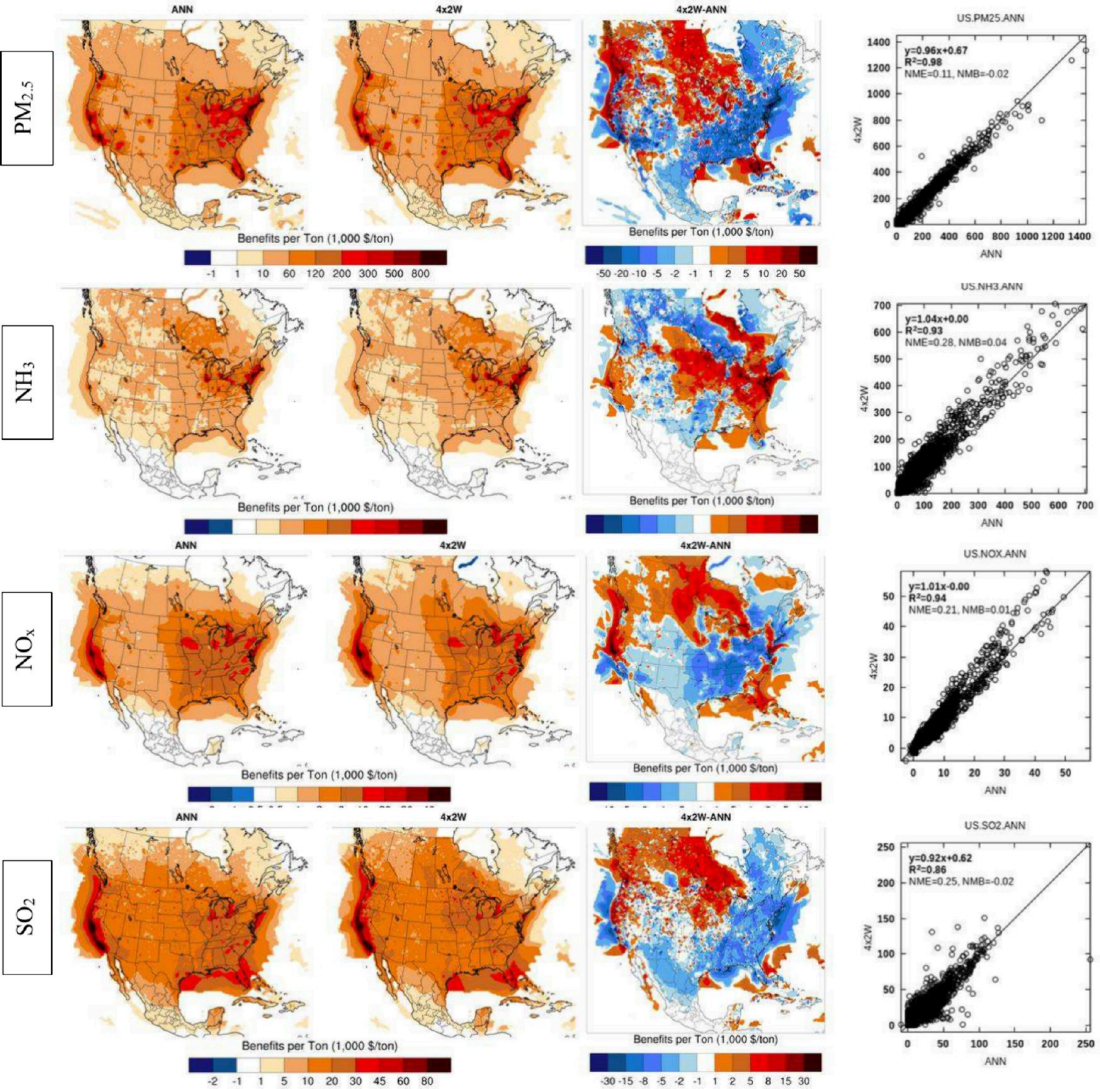
Comparison of annual surface BPTs from episodic (2 weeks per season; 4x2W) and full year (ANN) simulations at 36 km resolution. Normalized mean bias (NMB) and errors (NME) associated with episodic representation are shown for each species. Also shown (3rd column) are differences between episodic and annual BPTs (episodic – Annual).

Despite our detailed approach to selecting representative episodes for the U.S., using a short period in lieu of a full season will introduce errors and uncertainties in the estimated BPTs. Our 36 km episodic simulations produce BPTs reasonably similar to those of full-year simulations for the U.S. without any significant bias (Figure 2). BPTs for all species show good agreement between episodic and full-year simulations, but agreement is strongest for primary PM_2.5_ emissions and weaker for precursor species. Our results^21^ suggest that primary PM_2.5_ emissions account for 71% of the total burden in the U.S., and therefore, their strong representation through episodic simulations is reassuring.

Episode selection was conducted on a national scale. As a result, the episodes appear to be representative nationwide, but they show various levels of regional bias, as seen in the difference plots in Figure 2. While these regional biases for BPTs of various species are generally small in comparison to the magnitude of BPTs (note the scales for BPTs and difference plots), they appear to be structured. For instance, in much of the Eastern U.S., episodic simulations are biased low for primary PM_2.5_ and NOx BPTs but biased high for ammonia. NOx BPTs in particular exhibit regional features as they are biased low in episodic simulation for much of the U.S. but biased high for emissions in most coastal areas and Western Canada. Overall, the selected episodes show the lowest representativeness and highest errors for SO_2_ BPTs, with a low bias in much of the Eastern U.S. Finally, we note that our episode selection approach aimed to minimize the bias in estimated BPTs and burdens, and therefore, it is likely to be more representative of areas with higher BPT and burdens.

The implied assumption in our evaluation of errors from episodic simulations is that temporal trends and patterns at 36 km are similar to those of 12 km simulations that are the basis for our BPT evaluations. We further examine this assumption in our sensitivity analysis of horizontal resolution below and find it to be a justifiable assumption.

### 3.2 Choice of CRF

**Figure 3:**
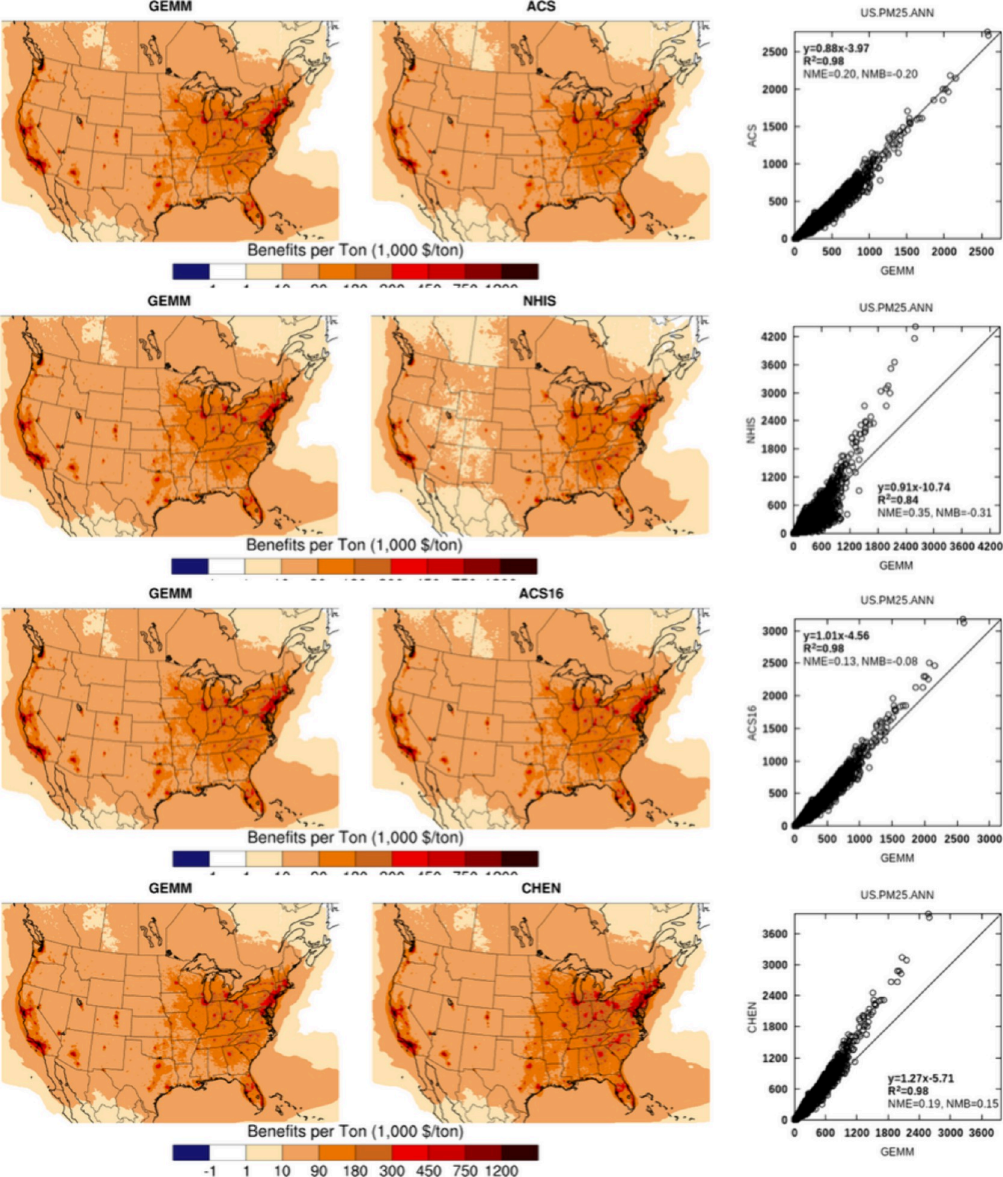
Comparison between surface primary PM_2.5_ BPTs calculated based on various CRFs for the U.S. Results for other species are shown in Figures A2–A4 in SI.

U.S. BPTs based on the GEMM CRF are compared with those from the two ACS-CPS-II cohorts (ACS-09 and ACS-16), NHIS, and CHEN as calculated through episodic simulations at 12 km. Figure 3 shows these comparisons for primary PM_2.5_ emissions, while those for precursor species are shown in the Supporting Information (Appendix A, Figures A2–A4). Overall, GEMM BPTs are consistently larger than ACS-09 BPTs, with a fairly consistent ratio of approximately 1.1–1.2 for different species. However, GEMM BPTs show better agreement with the more recent log–linear CRFs of ACS-16 and CHEN. On the other hand, comparison of GEMM and NHIS BPTs show more spread and bifurcation, partly due to the different curvatures (sublinear vs superlinear) of these CRFs.

A closer comparison of NHIS, GEMM, and ACS-09 provides insight on the role that the shape of CRF plays in BPT magnitudes, as in those we have three different shapes of CRFs represented. GEMM is a sublinear (concave) CRF, while NHIS has a superlinear (convex) shape and ACS-09 is a linear (i.e., log–linear) CRF. The adjoint system of equations is driven by the adjoint forcing terms, which are proportional to the slope of the population attributable fraction (PAF) for each CRF (Appendix A, Figure A1). The magnitude of the forcing term for nonlinear CRFs is a function of annual average concentrations of PM_2.5_ at each location. As similar atmospheric conditions apply to simulations for all 3 CRFs, different forcing terms are the main source of discrepancy between the estimated BPTs. For much of the lower range of concentrations (<10 μg/m^3^), GEMM has a larger forcing term than ACS-09 and NHIS (Figure A1) and is expected to produce larger BPTs. At annual average concentrations above 10 μg/m^3^, the NHIS forcing term exceeds that of GEMM, resulting in larger NHIS BPT estimates. Therefore, within the practical range of 2016 PM_2.5_ concentrations in the U.S., forcing terms may be larger for either of the GEMM or NHIS CRFs at different locations, leading to a less consistent relationship between those two sets of BPTs. As a log–linear CRF, the ACS-09 forcing changes slightly with concentrations and exceeds the GEMM forcing at concentrations above approximately 25 μg/m^3^. As annual average concentrations rarely exceeded that level for the U.S. in 2016, ACS-09 BPTs are consistently lower than GEMM. ACS-16 and CHEN have proportionally larger effect estimates than ACS-09 and therefore show similar behavior but at different thresholds. Finally, for each CRF, the same forcing affects BPTs of all species; however, different species undergo different chemical or thermodynamic transformation pathways, resulting in further interspecies differences.

Variability across BPTs from different CRFs provides a measure of uncertainties associated with the choice of CRF. This variability results in Coefficients of Variation (COV) that range between 15% and 100% for different emitted species, depending on the size of BPT (Appendix A, Figure A5); in general, COV is smaller in areas with larger BPTs. For areas with larger BPTs, the floor of variability across different CRFs, expressed as the COV, is approximately 15% for primary PM_2.5_ and NH_3_, 20% for NOx, and approximately 30% for SO_2_. It is important to note that this uncertainty does not include, and is in addition to, the statistical model uncertainty in each individual CRF, expressed as standard errors and confidence intervals for parametric estimates within each model. While for nonlinear CRFs propagating statistical uncertainty through BPT calculations is challenging, for log–linear CRFs, a measure of uncertainty equivalent to COV can be inferred from the reported confidence intervals for effect estimates. For the three log–linear CRFs covered here, ACS-09, ACS-16, and CHEN, this approximation amounts to 30%, 10%, and 13% of statistical uncertainty in those individual CRFs, respectively. Therefore, our results suggest that the uncertainty introduced by the choice of CRF is at least as large and possibly larger (depending on species) than the uncertainty in each epidemiological model itself. The impact of the choice of CRF on BPT estimates also translates into variability in the estimated burdens (Table 1), with an average COV in the estimated burden of about 16%.

**Table 1:** Impact of the Choice of CRF on the Estimated U.S. Burden ($Billion)^a^

	ACS-09	ACS-16	CHEN	GEMM	NHIS	COV (%)
PM_2.5_	507	584	730	585	511	15
NH_3_	111	128	160	129	107	16
NOx	37	43	54	43	37	16
SO_2_	41	48	60	48	39	17
Total	696	803	1004	805	694	16

a Burdens are shown for all U.S. emissions.

### 3.3 Model Resolution

**Figure 4:**
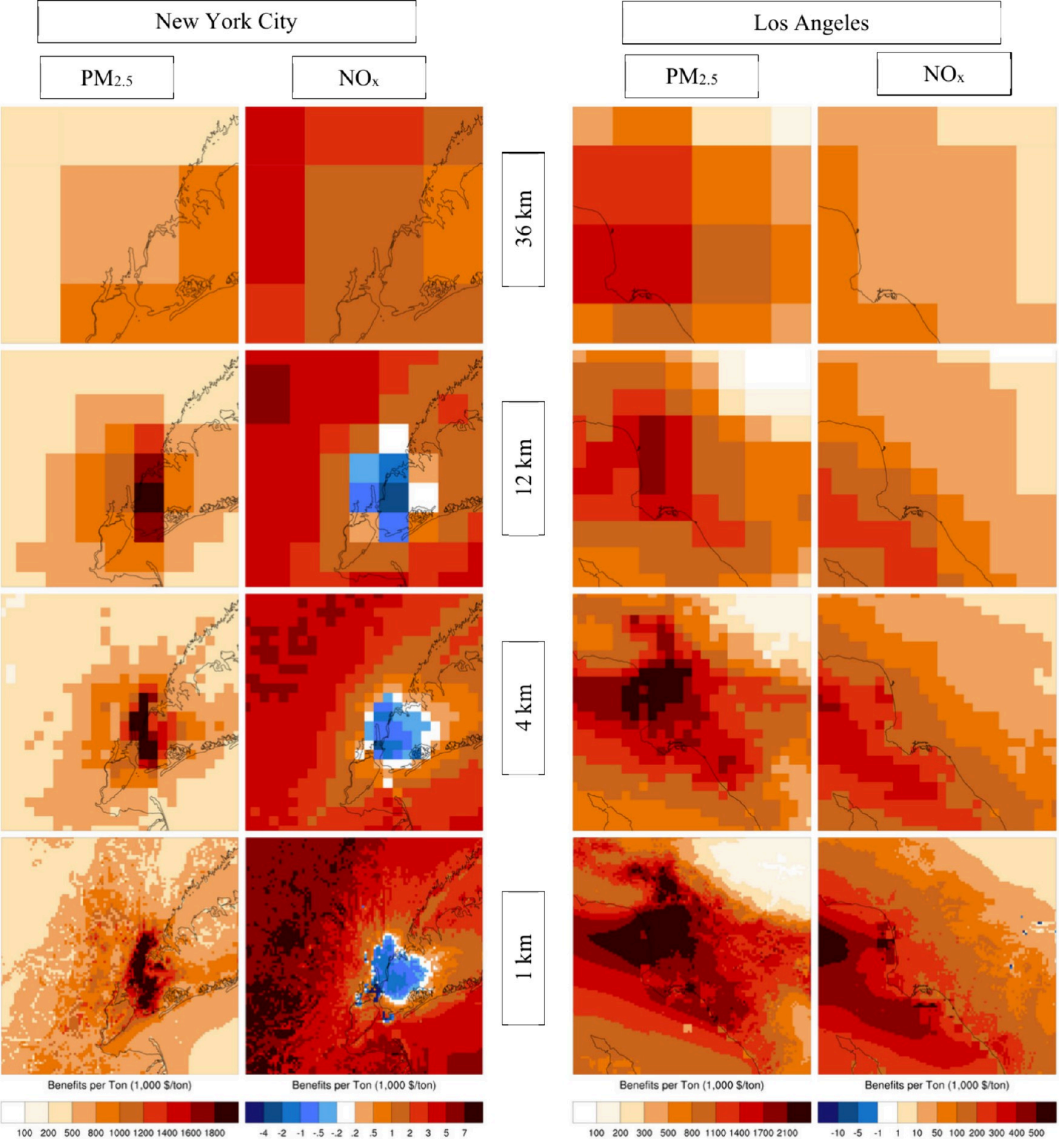
Summertime surface BPTs for PM_2.5_ and NOx emissions at 36, 12, 4, and 1 km resolutions for LA and NYC. BPTs for SO_2_ and NH_3_ emissions appear in Figure B1.

**Figure 5:**
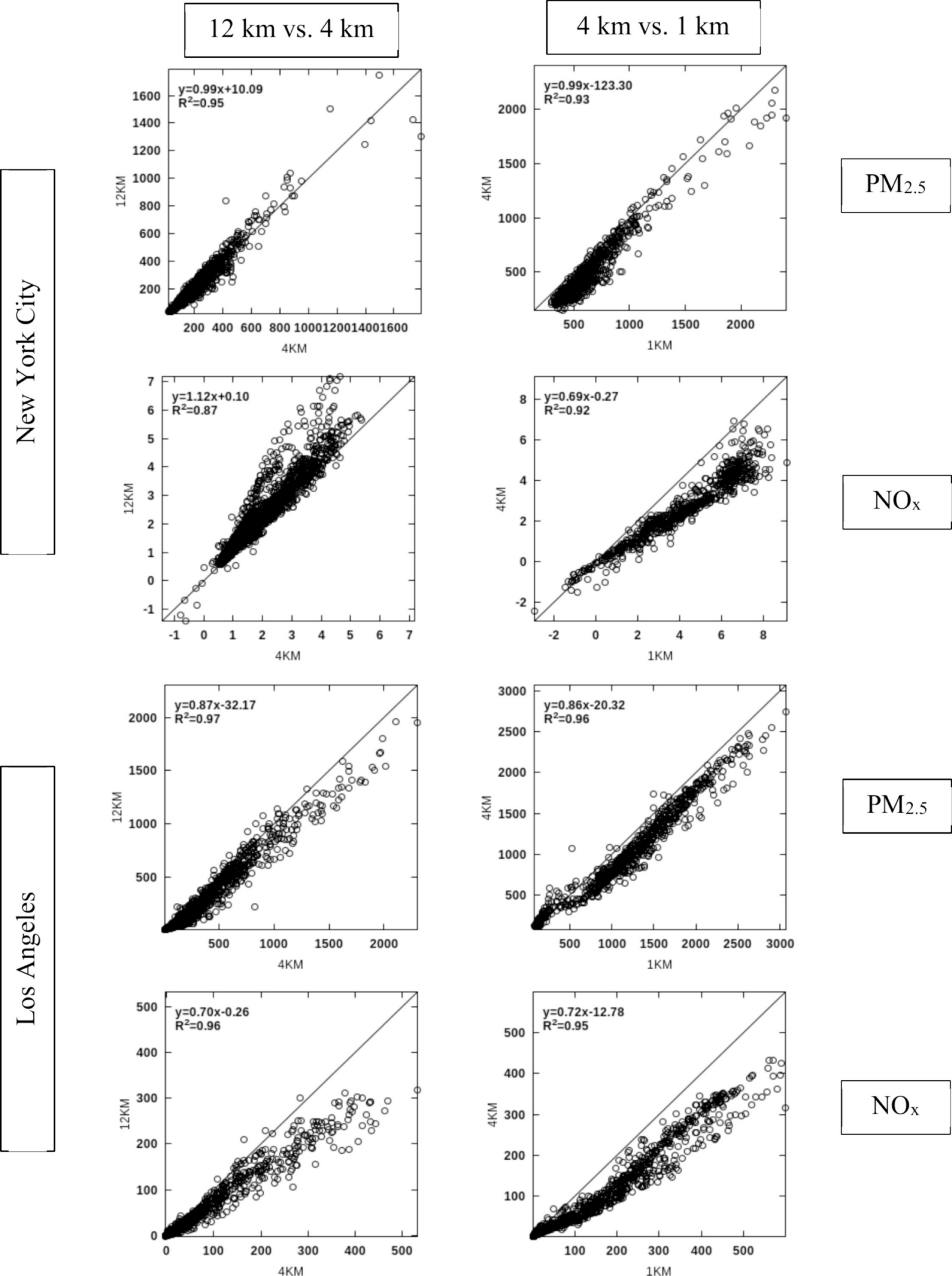
Comparison between 12 and 4 km (left panels) and 4 and 1 km (right panels) BPTs over LA and NYC for primary PM_2.5_ and NOx emissions. The same plots for NOx and SO2 emissions are shown in Figure B2. Comparisons are made between coarser resolution BPTs (e.g., 4 km) and aggregated values from finer resolution (e.g., 1 km) in the coarser grid.

4 and 1 km simulations are conducted for the summer episode only, and therefore, the comparisons presented here use that season for 12 and 36 km resolutions as well. While there is general consistency between the BPTs at different resolutions, there is a progression toward more detail and larger BPTs at higher resolutions (Figure 4 for PM_2.5_ and NOx and Figure B1 in Appendix B for SO_2_ and NH_3_). 36 km BPTs are consistent with 12 km results at the continental scale, which supports their use in episode selection. However, 36 km results appear inadequate in resolving BPTs in urban settings such as Los Angeles and New York City. 12 km BPTs appear to be consistent with higher resolution results over these two domains (Figure 5 and Figure B2), even though for most species they underestimate the BPTs at hot spots, particularly in Los Angeles, and for precursors of inorganic PM_2.5_.

Variability across BPTs at various resolutions also leads to differences in burden estimates (Table 2), where burden is defined, to a first-order approximation, as the product of BPTs and emissions at each location. In NYC, there is good consistency between resolutions for all primary and precursor emissions, resulting in consistent burden estimates. In Los Angeles, however, there is a substantial difference between the spatial distribution of 12 and 1 km BPTs, as well as the estimated burden for precursor emissions, particularly for NOx and NH_3_. For both Los Angeles and New York City, 1 km simulation can resolve areas of negative NOx BPTs that do not appear at coarser resolutions.

**Table 2:** Burden Estimates Based on Location-Specific BPTs at Various Resolutions for LA and NYC

	Los Angeles ($ Billion)				New York City ($ Billion)			
Species	36 km	12 km	4 km	1 km	36 km	12 km	4 km	1 km
PM_2.5_	21.6	21.1	25.3	29.6	12.1	18.4	17.3	19.3
NH_3_	3.1	4.3	6.4	13.6	0.3	0.6	0.7	0.9
NO_*x*_	2.7	5.1	8.3	13.7	0.2	0.1	0.1	0.2
SO_2_	0.7	1.4	1.2	1.8	0.3	0.5	0.5	0.7
Total	28.0	31.9	41.2	58.7	12.9	19.6	18.7	21.1

The literature on the impact of CTM grid resolution on PM_2.5_ burden is limited and somewhat unsettled. Punger et al.^32^ found higher U.S. burdens at lower resolution (36 km) than at higher resolution (12 km), but regridding their 12 km results, they found lower burdens at coarser resolutions used in global CTMs. Li et al.^33^ reported higher burdens at finer resolutions of a global model from direct simulations and regridded results. Similarly, Paolella et al.^34^ found higher population weighted exposures at higher resolutions when using an RCM. On the other hand, Thompson et al.^35^ found PM_2.5_ burdens to be rather insensitive to grid resolution (36, 12, and 4 km) in 9 urban areas in the Eastern U.S. Our results are unique as they explore much finer resolutions than previous studies, but they show a general trend toward higher burdens in finer resolutions similar to Li et al.^33^ and Paolella et al.,^34^ while our NYC results also conform with the findings of Thompson et al.^35^ in showing low sensitivity to resolution in Eastern U.S. cities.

**Figure 6:**
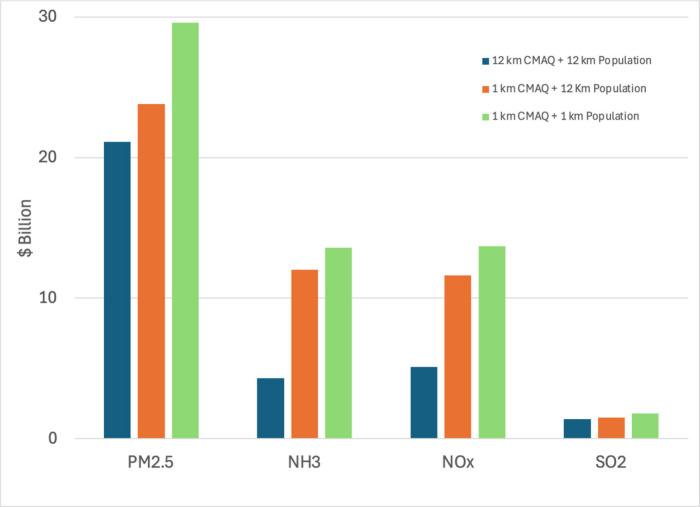
Impact of finer CTM and/or finer population representation on PM_2.5_ burdens in Los Angeles.

To further explore the behavior observed in Los Angeles, we conduct 1 km simulations with 12 km population distributions to help differentiate between the impact of atmospheric representation at higher resolution and better representation of the population distribution. We compare (Figure 6) a simulation with coarse grid and coarse population representation (i.e., 12 km simulations in Table 2), one with coarse population distribution but finely resolved CTM, and one where both the CTM and population are represented at high resolution (i.e., 1 km simulation in Table 2). For primary PM_2.5_ emissions, the majority of the higher burden at the finer resolution is due to the better resolved population distribution. This is expected because, for primary PM_2.5_ emissions, locations of high BPT and high emissions are often coincident with high populations and a better resolved representation of the atmospheric processes plays a less important role. For secondary inorganic precursors, the opposite appears to be true; finer representation of the chemical state appears to be more important. For these emitted precursor species, conversion to particle phase depends on the chemical state of the atmosphere. Their gas-to-particle conversion requires time, which may remove the formed particles from higher population and exposure. As higher resolution simulations reduce artificial dilution of high concentrations in the model, finer resolutions can lead to higher efficiency in gas-to-particle conversion. As a result, BPTs of NOx, NH_3_, and SO_2_ are more influenced by the higher CTM resolution than the more finely resolved population. We note that our higher resolution simulations also embed the impact of better resolved meteorology which may be significant for certain domains. The difference in the impact of resolution between Los Angeles and NYC may then be due to the differences in heterogeneities of population distribution and chemical state between the two domains.

### 3.4 Sensitivity to Emission Levels

**Figure 7:**
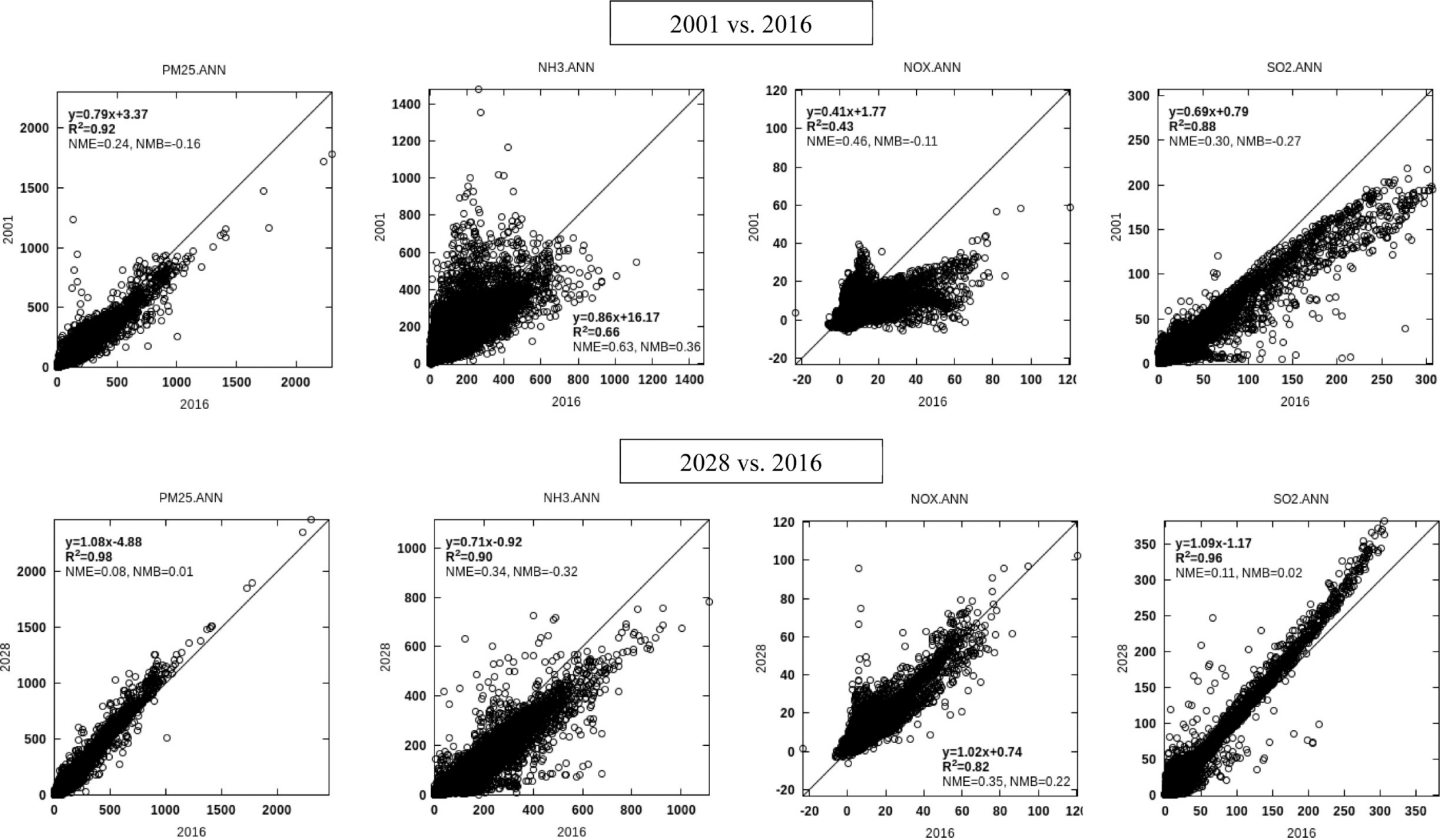
Comparison of BPT estimates between past (2001), present (2016), and future (2028) emissions levels.

Comparison of 2016 BPTs with those calculated with past and future inventories reveals interesting patterns (Figure 7). Our findings are consistent with those from Holt et al.^36^ who found larger source impacts for SO_2_ and NO_*x*_ and reduced source impact for ammonia in lower emission cases. While there are notable differences between 2001 and 2016 BPTs, there is a higher level of consistency between 2016 and 2028 estimates. This is expected as the change in North American emissions, and the resulting atmospheric composition in the 2001–2016 period is larger than the modeled changes during the 2016–2028 period. This is particularly true for changes in SO_2_ emissions and concentrations which greatly affects the availability of ammonia to combine with nitric acid to form aerosol nitrate. Of note, BPTs of primary PM_2.5_ emissions and SO_2_ (which typically has a near-linear oxidation pathway to sulfate) remain relatively stable between 2001, 2016, and 2028, as they are mainly affected by the epidemiological nonlinearities. These BPTs increase with reducing PM_2.5_ concentrations due to the sublinear form of GEMM, resulting in increased BPTs in later years and a cleaner atmosphere. In the case of SO_2_, and particularly from 2001 to 2016, reduced SO_2_ availability would also increase its conversion efficiency to sulfate due to reduced competition for oxidants (e.g., hydrogen peroxide in the aqueous phase). Reduced SO_2_ availability greatly impacts nitrate formation, as NO_*x*_ becomes more likely to form nitrate in 2016 than 2001, resulting in larger NO_*x*_ BPTs. Overall, it appears that, as the rate of change in atmospheric composition and, in particular, SO_2_ concentrations stabilize into the future, BPTs would also become more robust estimates for future scenarios.

## 4 Conclusions

Our analysis of the impact of inventory level suggests consistent estimates for primary PM_2.5_ BPTs but shows rather significant scatter between 2001 and 2016. While this difference partially diminishes between years 2016 and 2028, it highlights the need to use emissions inventories that are up-to-date and appropriate for the analysis time frame. This analysis also sheds light on the applicability and/or limitation of BPTs as a linear and slope-based approximation to source impacts in long-term, scenario-based projections. While one should remain mindful of the limitation of linear projections using BPTs for large-scale changes, it appears that cleaner atmosphere and reductions in SO_2_ availability will result in more robust BPT estimates across decadal time scales.

The sensitivity of source impact estimates to model resolution remains an important area for future research, particularly in environmental justice applications. Our sensitivity analysis finds that much of the time a higher resolution leads to somewhat greater BPT estimates, but the bias was not large or consistent in the two metropolitan areas studied. Our results suggest that, for primary PM_2.5_ emissions, better resolved population distribution is more influential, while for precusor emissions of secondary inorganic PM_2.5_, more resolved representation of the chemical state and meteorology is of higher value. Some of the local features such as negative NOx BPTs in NYC can be captured only by full complexity models and at high resolution. While the use of coarser resolution at the continental level is a practical necessity for full complexity models, the consistently larger estimates at the higher resolution suggest that national-level estimates are likely to be underestimations.

This analysis also shows that a well-chosen set of shorter (e.g., 2-week) periods can capture seasonal and annual responses. However, the choice of episodes needs to account for capturing a range of model responses. This is an important finding, not only for adjoint-based modeling studies but also for other modeling applications as it can allow using simulations with much higher spatial resolution than annual or multiyear simulations at coarser resolutions. This can be important when population-based assessments are used, where population demographics are important. An adjoint model, due to its ability to capture the roles of both the spatial population distribution and nonlinearities in atmospheric transformations, can reveal spatial sensitivities and identify locations where higher resolution is most important.

Our sensitivity analysis to the choice of CRF finds generally consistent estimates across epidemiological models of different shapes and cohorts. This is expected, as in the current range of PM_2.5_ concentrations in the U.S., all the CRFs produce comparable responses to reduction in exposures (see Figure A5 in Appendix A). The largest differences are found between the NHIS and GEMM models, with GEMM generally higher at lower BPT estimates while NHIS is higher at higher BPT ranges; an expected behavior for a sublinear CRF in GEMM compared to a superlinear CRF such as NHIS. The comparison between GEMM and the linear models was more consistent; although in a cleaner atmosphere, a sublinear CRF will likely produce larger source impacts in the future.

Although the impact of modeling choices investigated here are specific to an adjoint modeling study,^21^ the findings are more broadly applicable to other studies, as they suggest benefits from providing spatial detail and the use of a full-complexity model that can capture chemical nonlinearities. Our analysis of the sensitivities of the BPTs to the modeling approach (in both air quality and epidemiological models) shows a range of responses, varying by the pollutant and location. While our study is not a formal uncertainty analysis, our sensitivity analyses provides a semiquantitative measure of uncertainties arising from various aspects of study design. This level of detail can provide insight into the type of information that is most critical for conducting studies linking control measures to health benefits. In particular, the use of an adjoint model that offers spatially detailed sensitivities provides unique information on what locations may have the greatest potential for errors in such analyses.

## Data Availability

BPT estimates (surface and aloft) for GEMM and other CRFs for Canada and the U.S. are available at 10.5683/SP3/DTS44O.

## Supporting Information Available

Additional details of CRFs; BPT comparisons across CRFs for additional species; BPTs at different resolutions for SO_2_ and NH_3_ (PDF)

## References

[ref1] Fann N.; Fulcher C. M.; Hubbell B. J. The Influence of Location, Source, and Emission Type in Estimates of the Human Health Benefits of Reducing a Ton of Air Pollution. Air Qual Atmos Health 2009, 2 (3), 169–176. 10.1007/s11869-009-0044-0. 19890404 PMC2770129

[ref2] Fann N.; Baker K. R.; Fulcher C. M. Characterizing the PM2.5-Related Health Benefits of Emission Reductions for 17 Industrial, Area and Mobile Emission Sectors across the U.S.. Environment International 2012, 49, 141–151. 10.1016/j.envint.2012.08.017. 23022875

[ref3] Wolfe P.; Davidson K.; Fulcher C.; Fann N.; Zawacki M.; Baker K. R. Monetized Health Benefits Attributable to Mobile Source Emission Reductions across the United States in 2025. Science of The Total Environment 2019, 650, 2490–2498. 10.1016/j.scitotenv.2018.09.273. 30296769 PMC7259328

[ref4] Health Canada. Health Benefits Per Tonne of Air Pollutant Emissions Reduction: Region-, Sector-, and Pollutant-Specific Estimates for Two Canadian Regions; Health Canada, 2022.

[ref5] Hakami A.; Seinfeld J. H.; Chai T.; Tang Y.; Carmichael G. R.; Sandu A. Adjoint Sensitivity Analysis of Ozone Nonattainment over the Continental United States. Environ. Sci. Technol. 2006, 40 (12), 3855–3864. 10.1021/es052135g. 16830553

[ref6] Heo J.; Adams P. J.; Gao H. O. Public Health Costs of Primary PM2.5 and Inorganic PM2.5 Precursor Emissions in the United States. Environ. Sci. Technol. 2016, 50 (11), 6061–6070. 10.1021/acs.est.5b06125. 27153150

[ref7] Heo J.; Adams P. J.; Gao H. O. Reduced-Form Modeling of Public Health Impacts of Inorganic PM2.5 and Precursor Emissions. Atmos. Environ. 2016, 137, 80–89. 10.1016/j.atmosenv.2016.04.026.

[ref8] Muller N. Z.; Mendelsohn R.; Nordhaus W. Environmental Accounting for Pollution in the United States Economy. American Economic Review 2011, 101 (5), 1649–1675. 10.1257/aer.101.5.1649.

[ref9] Tessum C. W.; Hill J. D.; Marshall J. D. InMAP: A Model for Air Pollution Interventions. PLoS One 2017, 12 (4), e0176131 10.1371/journal.pone.0176131. 28423049 PMC5397056

[ref10] Henneman L. R. F.; Dedoussi I. C.; Casey J. A.; Choirat C.; Barrett S. R. H.; Zigler C. M. Comparisons of Simple and Complex Methods for Quantifying Exposure to Individual Point Source Air Pollution Emissions. J. Expo Sci. Environ. Epidemiol 2021, 31 (4), 654–663. 10.1038/s41370-020-0219-1. 32203059 PMC7494583

[ref11] Simon H.; Baker K. R.; Sellers J.; Amend M.; Penn S. L.; Bankert J.; Chan E. A. W.; Fann N.; Jang C.; McKinley G.; Zawacki M.; Roman H. Evaluating Reduced-Form Modeling Tools for Simulating Ozone and PM2.5 Monetized Health Impacts. Environ. Sci.: Atmos. 2023, 3, 1306–1318. 10.1039/D3EA00092C. PMC10425884 37590244

[ref12] Gilmore E. A.; Heo J.; Muller N. Z.; Tessum C. W.; Hill J. D.; Marshall J. D.; Adams P. J. An Inter-Comparison of the Social Costs of Air Quality from Reduced-Complexity Models. Environ. Res. Lett. 2019, 14 (7), 074016 10.1088/1748-9326/ab1ab5.

[ref13] Sandu A.; Daescu D. N.; Carmichael G. R.; Chai T. Adjoint Sensitivity Analysis of Regional Air Quality Models. J. Comput. Phys. 2005, 204 (1), 222–252. 10.1016/j.jcp.2004.10.011.

[ref14] Hakami A.; Henze D. K.; Seinfeld J. H.; Singh K.; Sandu A.; Kim S.; Byun; Li Q. The Adjoint of CMAQ. Environ. Sci. Technol. 2007, 41 (22), 7807–7817. 10.1021/es070944p. 18075092

[ref15] Henze D. K.; Hakami A.; Seinfeld J. H. Development of the Adjoint of GEOS-Chem. Atmospheric Chemistry and Physics 2007, 7 (9), 2413–2433. 10.5194/acp-7-2413-2007.

[ref16] Zhao S.; Russell M. G.; Hakami A.; Capps S. L.; Turner M. D.; Henze D. K.; Percell P. B.; Resler J.; Shen H.; Russell A. G.; Nenes A.; Pappin A. J.; Napelenok S. L.; Bash J. O.; Fahey K. M.; Carmichael G. R.; Stanier C. O.; Chai T. A Multiphase CMAQ Version 5.0 Adjoint. Geoscientific Model Development 2020, 13 (7), 2925–2944. 10.5194/gmd-13-2925-2020. 33343831 PMC7745733

[ref17] Pappin A. J.; Hakami A. Source Attribution of Health Benefits from Air Pollution Abatement in Canada and the United States: An Adjoint Sensitivity Analysis. Environ. Health Perspect 2013, 121 (5), 572–579. 10.1289/ehp.1205561. 23434744 PMC3673189

[ref18] Turner M. D.; Henze D. K.; Hakami A.; Zhao S.; Resler J.; Carmichael G. R.; Stanier C. O.; Baek J.; Sandu A.; Russell A. G.; Nenes A.; Jeong G.-R.; Capps S. L.; Percell P. B.; Pinder R. W.; Napelenok S. L.; Bash J. O.; Chai T. Differences Between Magnitudes and Health Impacts of BC Emissions Across the United States Using 12 Km Scale Seasonal Source Apportionment. Environ. Sci. Technol. 2015, 49 (7), 4362–4371. 10.1021/es505968b. 25729920

[ref19] Koo J.; Wang Q.; Henze D. K.; Waitz I. A.; Barrett S. R. H. Spatial Sensitivities of Human Health Risk to Intercontinental and High-Altitude Pollution. Atmos. Environ. 2013, 71, 140–147. 10.1016/j.atmosenv.2013.01.025.

[ref20] Byun D.; Schere K. L. Review of the Governing Equations, Computational Algorithms, and Other Components of the Models-3 Community Multiscale Air Quality (CMAQ) Modeling System. Applied Mechanics Reviews 2006, 59 (2), 51–77. 10.1115/1.2128636.

[ref21] Zhao S.; Vasilakos P.; Alhusban A.; Oztaner B.; Krupnick A.; Chang H.; Russell A.; Hakami A. Spatiotemporally Detailed Quantification of Air Quality Benefits of Emissions Reductions - Part I: Benefit-per-Tonne Estimates for Canada and the US. ACS Environ. Sci. Technol. Air, 10.1021/acsestair.4c00127. PMC11474827 39417161

[ref22] Burnett R.; Chen H.; Szyszkowicz M.; Fann N.; Hubbell B.; Pope C. A.; Apte J. S.; Brauer M.; Cohen A.; Weichenthal S.; Coggins J.; Di Q.; Brunekreef B.; Frostad J.; Lim S. S.; Kan H.; Walker K. D.; Thurston G. D.; Hayes R. B.; Lim C. C.; Turner M. C.; Jerrett M.; Krewski D.; Gapstur S. M.; Diver W. R.; Ostro B.; Goldberg D.; Crouse D. L.; Martin R. V.; Peters P.; Pinault L.; Tjepkema M.; van Donkelaar A.; Villeneuve P. J.; Miller A. B.; Yin P.; Zhou M.; Wang L.; Janssen N. A. H.; Marra M.; Atkinson R. W.; Tsang H.; Quoc Thach T.; Cannon J. B.; Allen R. T.; Hart J. E.; Laden F.; Cesaroni G.; Forastiere F.; Weinmayr G.; Jaensch A.; Nagel G.; Concin H.; Spadaro J. V. Global Estimates of Mortality Associated with Long-Term Exposure to Outdoor Fine Particulate Matter. Proc. Natl. Acad. Sci. U. S. A. 2018, 115 (38), 9592–9597. 10.1073/pnas.1803222115. 30181279 PMC6156628

[ref23] Skamarock C.; Klemp B.; Dudhia J.; Gill O.; Barker D.; Duda G.; Huang X.; Wang W.; Powers G. A Description of the Advanced Research WRF Version 3. NCAR, 2008.

[ref24] US EPA. Technical Support Document: Preparation of Emissions Inventories for the Version 7.2, 2016 North American Emissions Modeling Platform; US EPA, 2019.

[ref25] Houyoux M.; Vukovich J. Updates to the Sparse Matrix Operator Kernel Emissions (SMOKE) Modeling System and Integration with Models-3; 1999.

[ref26] US EPA. Guidelines for Preparing Economic Analyses; US EPA, 2010.

[ref27] Krewski D.; Jerrett M.; Burnett R. T.; Ma R.; Hughes E.; Shi Y.; Turner M. C.; Pope C. A. III; Thurston G.; Calle E. E. Extended Follow-up and Spatial Analysis of the American Cancer Society Study Linking Particulate Air Pollution and Mortality; Health Effects Institute: Boston, MA, 2009; Vol. 140. 19627030

[ref28] Turner M. C.; Jerrett M.; Pope C. A.; Krewski D.; Gapstur S. M.; Diver W. R.; Beckerman B. S.; Marshall J. D.; Su J.; Crouse D. L.; Burnett R. T. Long-Term Ozone Exposure and Mortality in a Large Prospective Study. Am. J. Respir Crit Care Med. 2016, 193 (10), 1134–1142. 10.1164/rccm.201508-1633OC. 26680605 PMC4872664

[ref29] Chen J.; Hoek G. Long-Term Exposure to PM and All-Cause and Cause-Specific Mortality: A Systematic Review and Meta-Analysis. Environ. Int. 2020, 143, 105974 10.1016/j.envint.2020.105974. 32703584

[ref30] Pope C. A.; Lefler J. S.; Ezzati M.; Higbee J. D.; Marshall J. D.; Kim S.-Y.; Bechle M.; Gilliat K. S.; Vernon S. E.; Robinson A. L.; Burnett R. T. Mortality Risk and Fine Particulate Air Pollution in a Large, Representative Cohort of U.S. Adults. Environ. Health Perspect. 2019, 127 (7), 077007 10.1289/EHP4438. 31339350 PMC6792459

[ref31] Öztaner Y. B.; Soltanzadeh M.; Zhao S.; Hakami A. Health Benefits of Phasing out Coal-Fired Power Plants in Ontario, Alberta, and Canada. Atmos. Environ. 2024, 334, 120711 10.1016/j.atmosenv.2024.120711.

[ref32] Punger E. M.; West J. J. The Effect of Grid Resolution on Estimates of the Burden of Ozone and Fine Particulate Matter on Premature Mortality in the United States. Air Qual Atmos Health 2013, 6 (3), 563–573. 10.1007/s11869-013-0197-8. PMC3862082 24348882

[ref33] Li Y.; Henze D. K.; Jack D.; Kinney P. L. The Influence of Air Quality Model Resolution on Health Impact Assessment for Fine Particulate Matter and Its Components. Air Qual Atmos Health 2016, 9 (1), 51–68. 10.1007/s11869-015-0321-z. 28659994 PMC5484574

[ref34] Paolella D. A.; Tessum C. W.; Adams P. J.; Apte J. S.; Chambliss S.; Hill J.; Muller N. Z.; Marshall J. D. Effect of Model Spatial Resolution on Estimates of Fine Particulate Matter Exposure and Exposure Disparities in the United States. Environ. Sci. Technol. Lett. 2018, 5 (7), 436–441. 10.1021/acs.estlett.8b00279.

[ref35] Thompson T. M.; Rausch S.; Saari R. K.; Selin N. E. A Systems Approach to Evaluating the Air Quality Co-Benefits of US Carbon Policies. Nature Clim Change 2014, 4 (10), 917–923. 10.1038/nclimate2342.

[ref36] Holt J.; Selin N. E.; Solomon S. Changes in Inorganic Fine Particulate Matter Sensitivities to Precursors Due to Large-Scale US Emissions Reductions. Environ. Sci. Technol. 2015, 49 (8), 4834–4841. 10.1021/acs.est.5b00008. 25816113

