# Supplementary material for: Spatiotemporally Detailed Quantification of Air Quality Benefits of Emissions–Part II: Sensitivity to Study Parameters and Assumptions
Source: ACS EST Air. 2024 Aug 27;1(10):1227–38. doi: 10.1021/acsestair.4c00128 (PMC11474814; doi:10.1021/acsestair.4c00128)
Supplement: Supplementary file 1 — ea4c00128_si_001.pdf [file ea4c00128_si_001.pdf]

Supplementary Information

for

Spatiotemporally Detailed Quantification Of Air Quality Benefits Of Emissions – Part II: Sensitivity to Study Parameters and Assumptions

Amir Hakami[a], Shunliu Zhao[a], Petros Vasilakos[b#], Anas Alhusban[a], Yasar Burak Oztaner[a], Alan Krupnick[c], Howard Chang[d], Armistead Russell[b]*

[a]Department of Civil and Environmental Engineering, Carleton University, Ottawa, ON K1S 5B6, Canada

[b] School of Civil and Environmental Engineering, Georgia Institute of Technology, Atlanta, GA 30331, USA

[c] Resources For the Future, Washington , DC 20036, U.S.A.

[d] Emory University, Atlanta, GA 30322, U.S.A.

Appendix A: Concentration Response Functions

GEMM

GEMM is a pooled cohort informed by 41 individual cohorts from 16 countries, and covers the range of concentrations seen across the globe. The choice of GEMM allows for the use of a single model in both Canada and the U.S. GEMM predicts globally higher estimates of air pollution burden than the global burden of disease studies [1], but North American estimates that are more in line with previous studies. For BPT estimations we use GEMM effect estimates for noncommunicable diseases and lower respiratory infections (NCD+LRI) with population and baseline rates for adults 25 years and older. GEMM has a sublinear (concave) CRF, with increasing rate of reduction in (Hazard Ratio) HR at lower concentrations (Figure S1-a).

GEMM is formulated into the adjoint cost function based on the following equations (Burnett et al., 2018):

$$J = V_{SL} \sum_i M_{0,i} \times P_i (1 - e^{-\theta T(z)}),$$

where,

$$T(z) = \log\left(1 + \frac{z}{\alpha}\right) \omega(z),$$

$$\omega(z) = \frac{1}{1 + e^{-(z-\mu)/\nu}} \quad ,$$

$$HR = e^{\theta T(z)}$$

and

$$z = MAX(0, PM_{2.5} - cf)$$

$M_{0,i}$ and P_i are baseline mortality rates and population (age > 25), respectively, cf indicates counterfactual concentration, and *HR* is the hazard ratio from the model. The coefficients in the above equations have the following values[1]: θ = 0.1231, α = 1.5, μ = 10.4, ν = 25.9, and cf = 2.4 μg/m3. Population data is generated using respective census information in Canada and the U.S., and is mapped to various grid resolutions. Similarly, baseline rates are taken from BenMAP, and are mapped/aggregated onto appropriate horizontal resolution in various simulations.

Linear CRFs

Krewski et al.[2] provided a reanalysis of the extended ACS-CPS cohort of approximately 360,000 adults aged 30 years and older. They used a standard Cox proportional hazard model and also the random effects Cox model and provided their respective effect estimates for the national cohort, as well as estimates for two urban areas. This CRF is linear and its adjoint cost function has the following formulation:

$$J = V_{SL} \sum_i M_{0,i} \times P_i (1 - e^{-\beta \overline{C_i}})$$

where $\beta = 0.00583\ (\frac{\mu g}{m^3})^{-1}$ is the effect estimate from the random effects model and $\overline{C_i}$ is the average $PM_{2.5}$ concentration. We refer to BPTs from this CRF as ACS-09.

In addition to the 2009 reanalysis of the ACS cohort, we also use single pollutant ($PM_{2.5}$) model of the updated analysis [3] for our sensitivity analyses. For this updated study an effect estimate of $\beta = 0.00677\ (\frac{\mu g}{m^3})^{-1}$ is used. Given the near-linear form of the CRF, BPTs for this updated analysis of the ACS cohort, referred to as ACS-16, is estimated using a simple scaling of BPTs from Krewski et al [2].

Similarly, we also provide BPTs for a linear CRF from the meta-analysis of Chen and Hoek[4] (referred to as CHEN). We use the effect estimate of $\beta = 0.00862\ (\frac{\mu g}{m^3})^{-1}$ for the middle-age population of the cohorts used in the meta-analysis, as it more closely aligns with other cohorts used in our study.

NHIS

The NHIS study is from a cohort of approximately 1.5 million individuals aged 18 – 84 who conducted the survey between the years 1986 – 2014[5]. The size and longitudinal extent of the cohort allowed for a large number of deaths to be recorded in the cohort. The CRF for the NHIS cohort is superlinear (convex) unlike the GEMM model, indicating lower rate of change in HR with decreasing concentrations. The adjoint cost function based on the NHIS CRF can be written as [6]:

$$J = V_{SL} \sum_{i} M_{0,i} \times P_i(1 - e^{-\theta T_{NHIS}(z)}),$$

where,

$$T_{NHIS}(z) = \frac{z}{1 + e^{-(z-\mu)/v}} = \quad z\omega(z)$$

and, z = MAX(0,$PM_{2.5}$ − cf), cf = 2.5 μg/m3, θ = 0.011253, μ = 8.330415, ν = τ × RANGE, τ = 0.2, RANGE = 16.7. Note, the form of ω(z) is identical to that of the GEMM model.

(1) Burnett, R.; Chen, H.; Szyszkowicz, M.; Fann, N.; Hubbell, B.; Pope, C. A.; Apte, J. S.; Brauer, M.; Cohen, A.; Weichenthal, S.; Coggins, J.; Di, Q.; Brunekreef, B.; Frostad, J.; Lim, S. S.; Kan, H.; Walker, K. D.; Thurston, G. D.; Hayes, R. B.; Lim, C. C.; Turner, M. C.; Jerrett, M.; Krewski, D.; Gapstur, S. M.; Diver, W. R.; Ostro, B.; Goldberg, D.; Crouse, D. L.; Martin, R. V.; Peters, P.; Pinault, L.; Tjepkema, M.; van Donkelaar, A.; Villeneuve, P. J.; Miller, A. B.; Yin, P.; Zhou, M.; Wang, L.; Janssen, N. A. H.; Marra, M.; Atkinson, R. W.; Tsang, H.; Quoc Thach, T.; Cannon, J. B.; Allen, R. T.; Hart, J. E.; Laden, F.; Cesaroni, G.; Forastiere, F.; Weinmayr, G.; Jaensch, A.; Nagel, G.; Concin, H.; Spadaro, J. V. Global Estimates of Mortality Associated with Long-Term Exposure to Outdoor Fine Particulate Matter. *Proceedings of the National Academy of Sciences* **2018**, *115* (38), 9592–9597. https://doi.org/10.1073/pnas.1803222115.

(2) Krewski, D.; Jerrett, M.; Burnett, R. T.; Ma, R.; Hughes, E.; Shi, Y.; Turner, M. C.; Pope III, C. A.; Thurston, G.; Calle, E. E. *Extended Follow-up and Spatial Analysis of the American Cancer Society Study Linking Particulate Air Pollution and Mortality*; Health Effects Institute Boston, MA, 2009; Vol. 140.

(3) Turner, M. C.; Jerrett, M.; Pope, C. A.; Krewski, D.; Gapstur, S. M.; Diver, W. R.; Beckerman, B. S.; Marshall, J. D.; Su, J.; Crouse, D. L.; Burnett, R. T. Long-Term Ozone Exposure and Mortality in a Large Prospective Study. *Am J Respir Crit Care Med* **2016**, *193* (10), 1134–1142. https://doi.org/10.1164/rccm.201508-1633OC.

(4) Chen, J.; Hoek, G. Long-Term Exposure to PM and All-Cause and Cause-Specific Mortality: A Systematic Review and Meta-Analysis. *Environ Int* **2020**, *143*, 105974. https://doi.org/10.1016/j.envint.2020.105974.

(5) Pope, C. A.; Lefler, J. S.; Ezzati, M.; Higbee, J. D.; Marshall, J. D.; Kim, S.-Y.; Bechle, M.; Gilliat, K. S.; Vernon, S. E.; Robinson, A. L.; Burnett, R. T. Mortality Risk and Fine Particulate Air

Pollution in a Large, Representative Cohort of U.S. Adults. *Environmental Health Perspectives* **2019**, *127* (7), 077007. https://doi.org/10.1289/EHP4438.
(6) Burnett, R. T. Personal Communication., 2020.

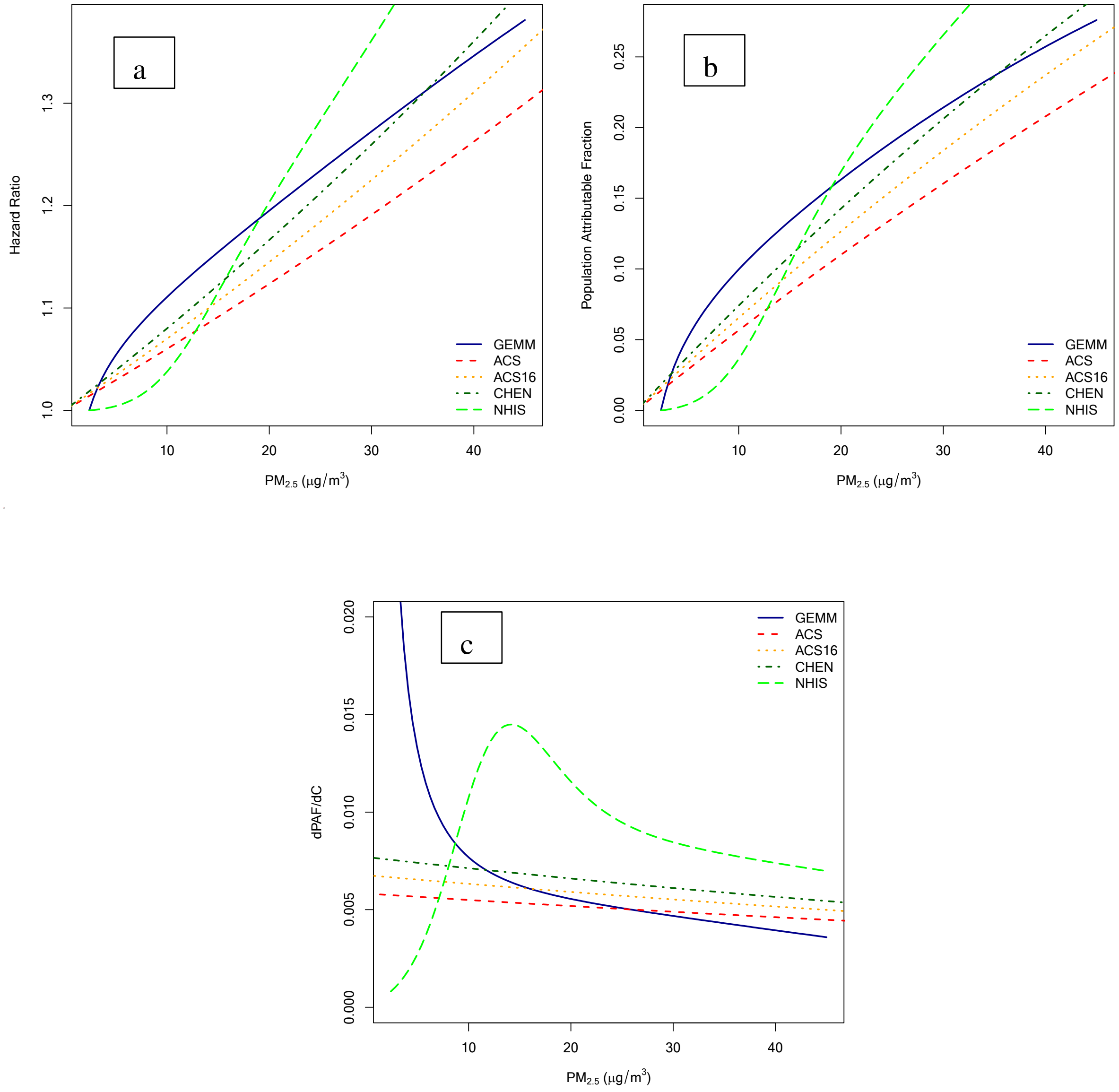

Figure A1: a) Hazard ratios (HR), b) population attributable fractions (PAF), and c) the slope of PAF with respect to concentrations.

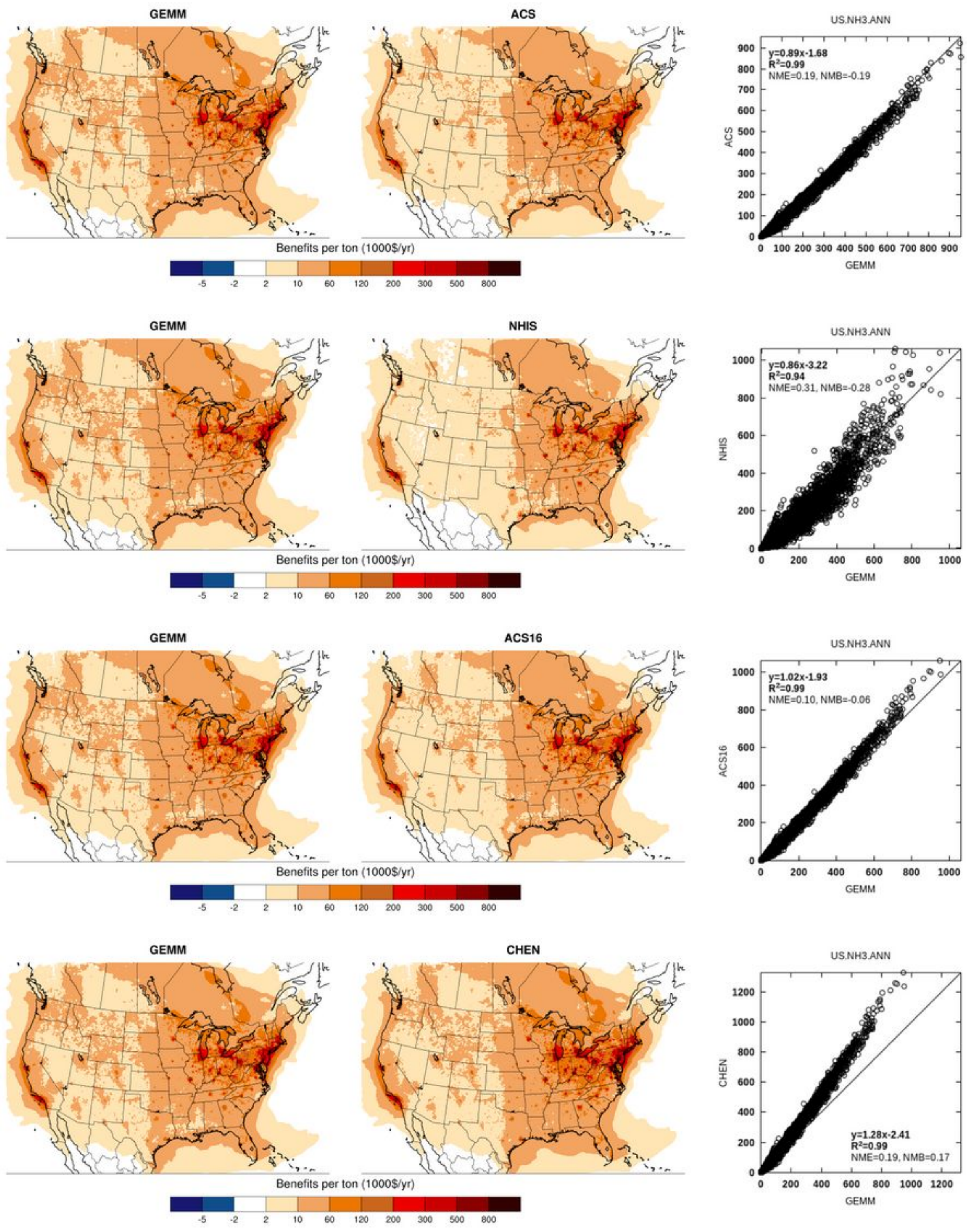

Figure A2: Comparison between surface BPTs calculated based on various CRFs for the U.S. for NH_3 emissions.

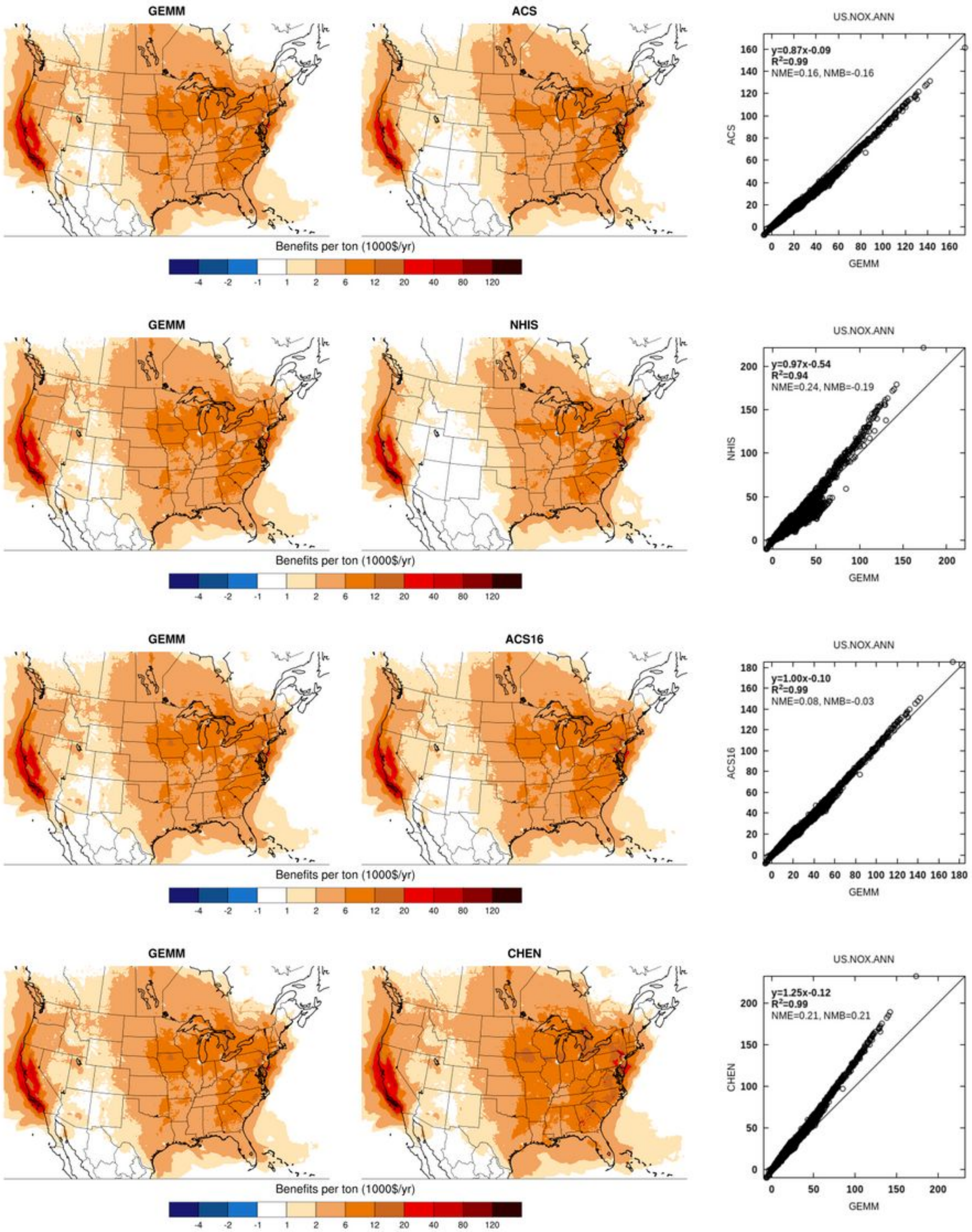

Figure A3: Comparison between surface BPTs calculated based on various CRFs for the U.S. for NO_x emissions.

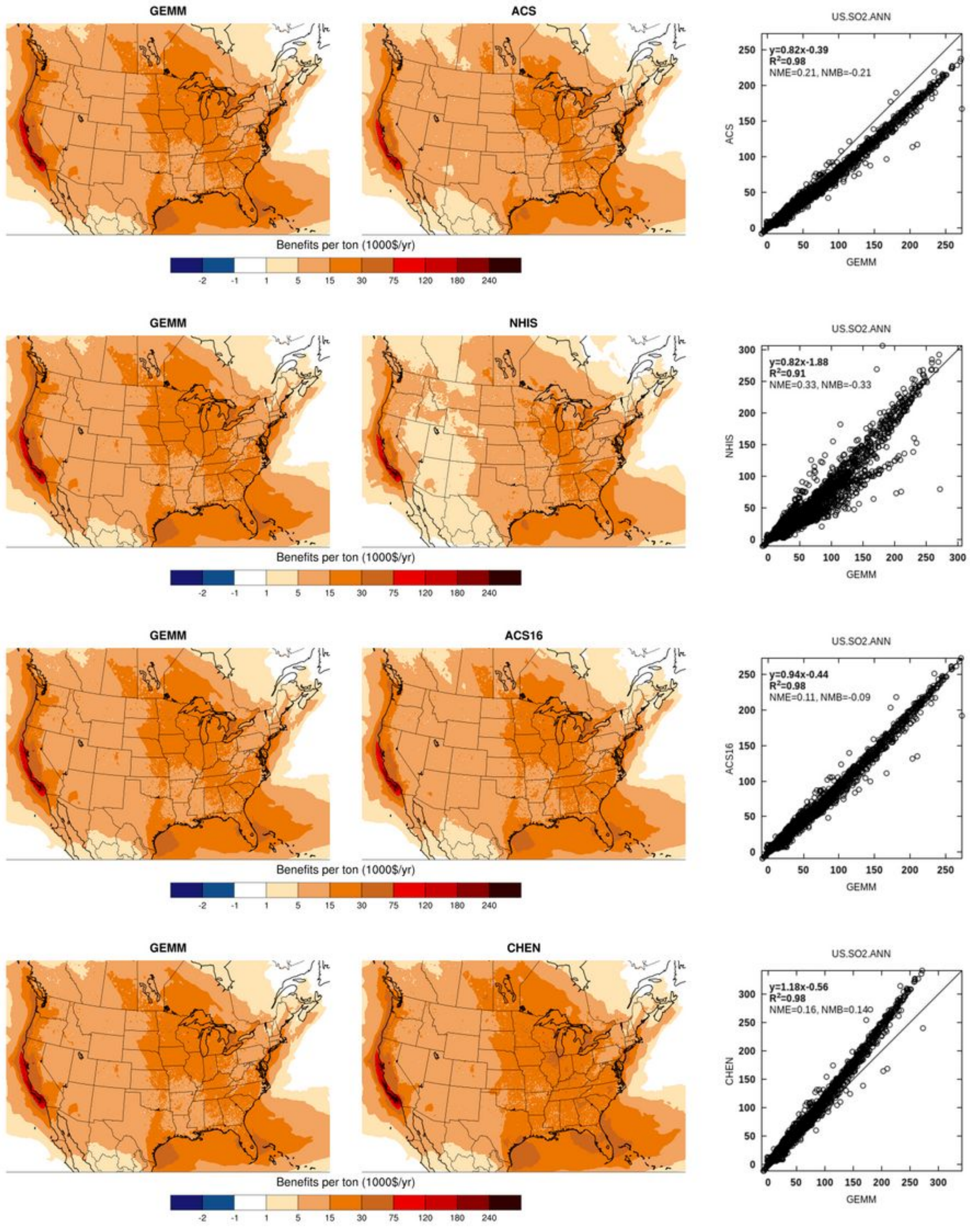

Figure A4: Comparison between surface BPTs calculated based on various CRFs for the U.S. for SO_2 emissions.

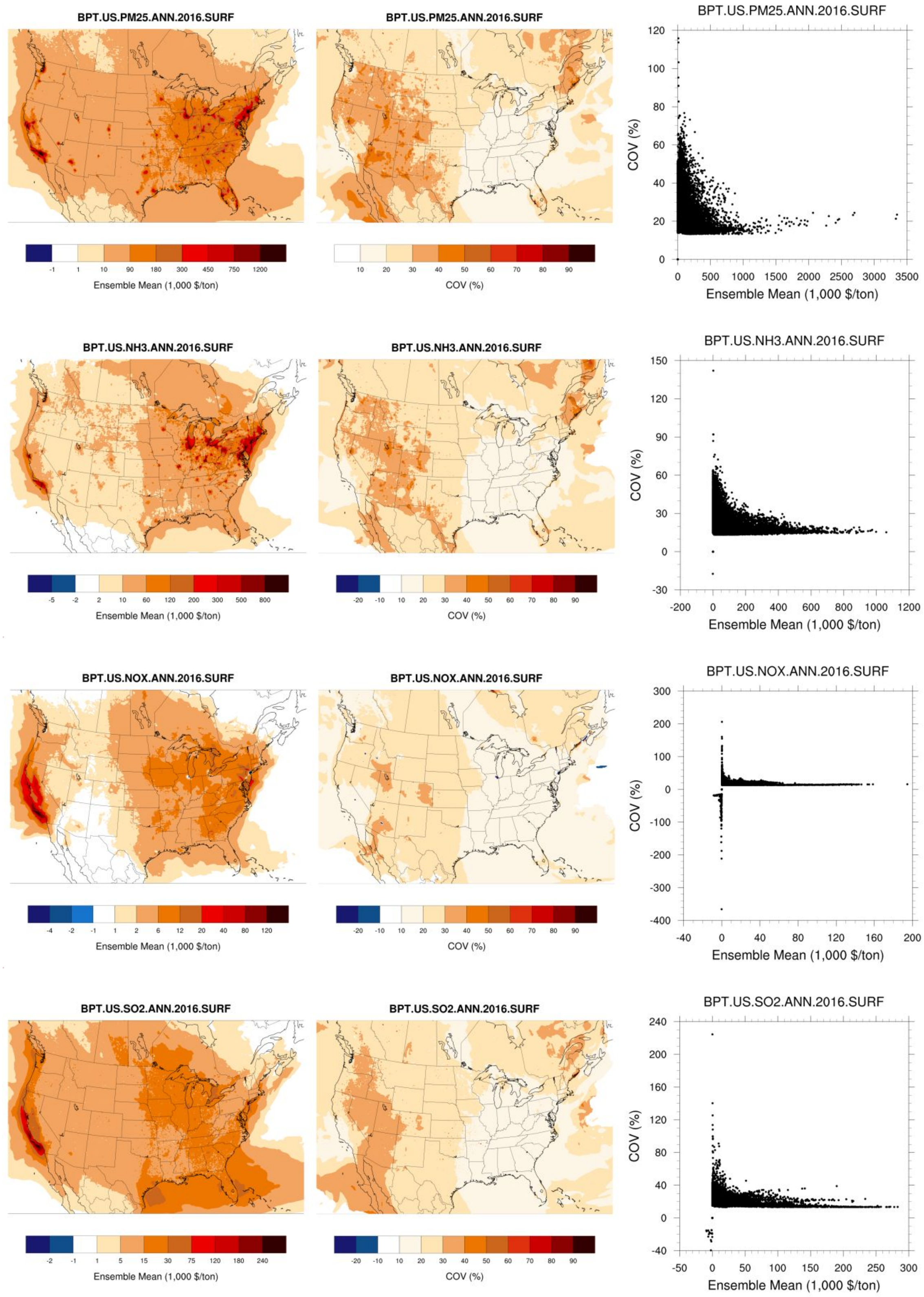

Figure A5: Mean and coefficient of variations (%) for the five BPT estimates. Scatter plots on the right panels show that coefficient of variation is smaller in areas with large BPTs.

Appendix B: Sensitivity to Resolution, NH_3 and SO_2 BPTs

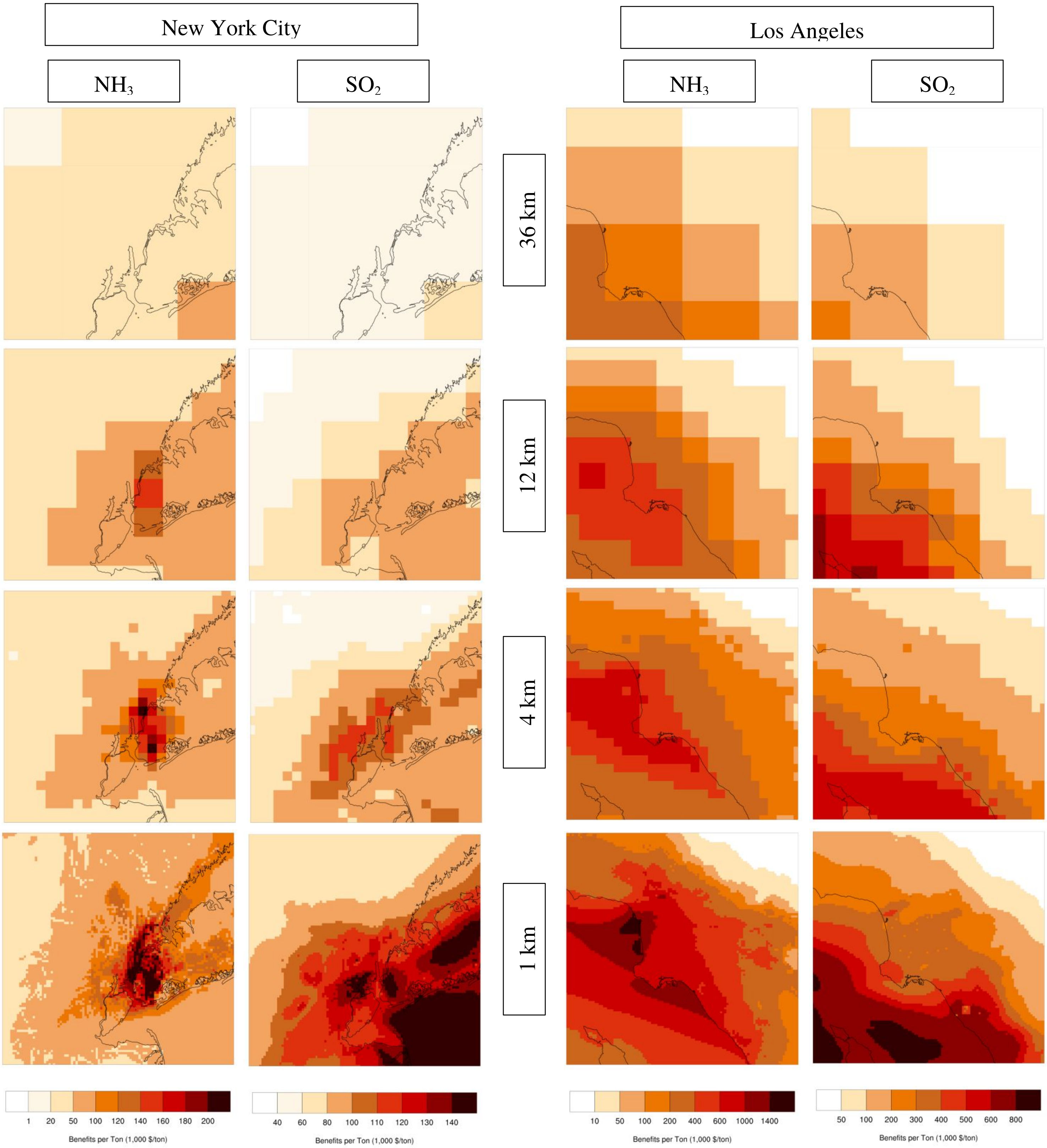

Figure B1. Summertime surface BPTs for SO_2 and NH_3 emissions at 36, 12, 4, and 1 km resolutions for LA and NYC.

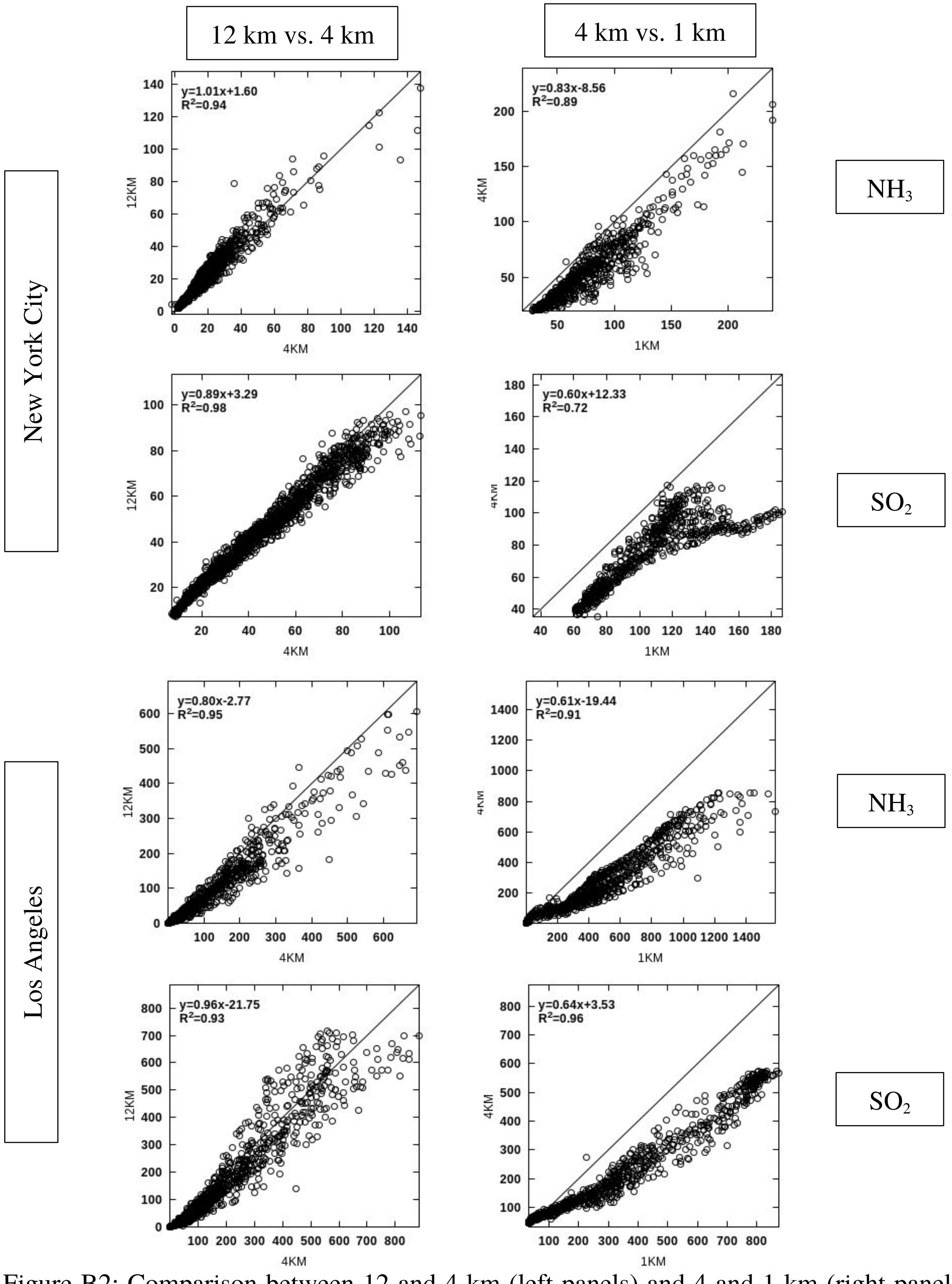

Figure B2: Comparison between 12 and 4 km (left panels) and 4 and 1 km (right panels) BPTs over LA and NYC for primary NH_3 and SO_2 emissions. Same plots for $PM_{2.5}$ and NO_x emissions are shown in Figure 4. Comparisons are made between coarser resolution values (e.g., 4 km) and aggregated values from finer resolution (e.g., 1 km) into the coarser grid.
